# Hand hygiene versus additional non-sterile gloves and gowns use to prevent sepsis in preterm infants colonized with multi-resistant drug bacteria: the study protocol of the cluster-randomized, cross-over, non-inferiority BALTIC trial

**DOI:** 10.1186/s40348-025-00192-2

**Published:** 2025-04-07

**Authors:** Kirstin Faust, Clara Haug, Julia Pagel, Reinhard Jensen, Anja Stein, Ursula Felderhoff-Müser, David Frommhold, Kirsten Brebach, Christian Wieg, Georg Hillebrand, Barbara Naust, Esther Schmidt, Lutz Koch, Susanne Schmidtke, Arne Simon, Michael Zemlin, Sascha Meyer, Christopher Scholzen, Natascha Köstlin-Gille, Christian Gille, Ann-Carolin Longardt, Manuela Kärlin, Mirjam Lusga, Wolfgang Göpel, Manuel Krone, Stefanie Kampmeier, Franziska Strecker, Dennis Nurjadi, Inke R. König, Egbert Herting, Jan Rupp, Christoph Härtel

**Affiliations:** 1 Department of Pediatrics, University Hospital Schleswig-Holstein, Lübeck, Germany; 2 German Center for Infection Research, Site Hamburg-Lübeck-Borstel-Riems, Hamburg, Germany; 3 Department of Pediatrics, University Hospital Hamburg-Eppendorf, Hamburg-Eppendorf, Germany; 4 Children’s Hospital Heide, Heide, Germany; 5 Department of Pediatrics I, Neonatology, University Hospital Essen, Essen, Germany; 6 Children’s Hospital Memmingen, Memmingen, Germany; 7 Children’s Hospital Aschaffenburg-Alzenau, Alzenau, Germany; 8 Children’s Hospital Itzehoe, Itzehoe, Germany; 9 Helios Children’s Hospital Schwerin, Schwerin, Germany; 10 Children’s Hospital Wilhelmstift Hamburg and Marienhospital Hamburg, Hamburg, Germany; 11 Neonatology, Asklepios Hospital Hamburg-Barmbek, Hamburg, Germany; 12 Department of Pediatric Hematology and Immunology, Saarland University Medical Center, Homburg, Germany; 13 Department for General Pediatrics and Neonatology, Saarland University Medical Center, 66421 Homburg, Germany; 14 SKK Klinikum, Franz-Lust Klinik für Kinder und Jugendliche, Karlsruhe, Germany; 15 Department of Neonatology, University Hospital Tübingen, Tübingen, Germany; 16 Department of Neonatology, University Hospital Heidelberg, Heidelberg, Germany; 17 Department of Pediatrics, University Hospital Schleswig-Holstein, Kiel, Germany; 18 Infection Control and Antimicrobial Stewardship Unit, University Hospital Würzburg, Würzburg, Germany; 19 Department of Infectious Diseases and Microbiology, University Hospital Schleswig-Holstein, Lübeck, Germany; 20 Institute of Medical Biometry and Statistics, University of Lübeck, Lübeck, Germany; 21 Department of Pediatrics, University Hospital Würzburg, Josef-Schneider-Strasse 2, D-97080 Würzburg, Germany

**Keywords:** Preterm infants, Sepsis, Multi-drug-resistant organisms, Hand hygiene, Gown, Non-sterile glove use

## Abstract

**Background:**

Infections are highly relevant for neonatal mortality and long-term morbidities in survivors. Therefore, it is an urgent need to optimize and evaluate infection prevention and control (IPC) strategies. Several infection outbreaks in German neonatal intensive care units (NICUs) required rapid responses by hospitals and improved future preparedness. As a consequence, German authorities recommended weekly colonization screening on NICUs. This screening aims to detect multidrug-resistant organisms (MDRO) and bacteria with high transmissibility. According to these guidelines, infants colonized with multiresistant gram-negative (MRGN) bacteria with in-vitro resistance to piperacillin and cephalosporins (2MRGN) should be cared wearing non-sterile gloves and gowns in addition to standard hygiene precautions. Whether these extended IPC measures have an individual benefit for infants or contribute to the prevention of infection outbreaks has not yet been scientifically proven. This study aims to evaluate the effect of hand desinfection as compared to hand desinfection + gloves and gowns (barrier care) for the care of 2MRGN colonized infants in NICUs on infection and transmission rates through a multicenter, cluster randomized controlled trial (BALTIC study, **Ba**rrier protection to **l**ower **t**ransmission and **i**nfection rates with Gram-negative 2-MRGN in preterm **c**hildren).

**Methods:**

12 participating NICUs were randomly allocated to two trial arms: receiving the intervention “standard precautions with a special focus on hand desinfection” or control (standard precautions “plus” barrier care) for the care of 2MRGN positive infants. Cross over was performed after 12 months for another 12 months per site. Primary outcome was the rate of healthcare-associated (HA) Gram-negative bloodstream infections. Secondary outcomes included transmission rate with screening relevant bacteria, overall rate of clinical and culture-proven infections, number of antibiotic cycles and desinfectant use. Regular trainings and hygiene audits are standardized co-interventions.

**Benchmarking results:**

According to our single center data, 9.3% of NICU-treated infants are colonized with 2MRGN during their hospital stay. BALTIC randomized the first center in October 2020 and finished data collection including close-out monitoring in January 2024. Data analysis will be completed in May 2025.

**Conclusions:**

BALTIC should contribute to better evidence on the effectiveness of hand desinfection and extended barrier precautions in critically ill newborns. Further benefits include comprehensive multi-center data collection on MDRO colonization dynamics, an improved awareness on IPC strategies and establishment of network platforms including antimicrobial stewardship programs.

**Supplementary Information:**

The online version contains supplementary material available at 10.1186/s40348-025-00192-2.

## Background and rationale

Advances in perinatal medicine and neonatology have led to significantly improved survival rates for extremely immature premature babies. Modern care of very low birthweight infants (VLBWI) and critically ill newborns is well standardized in neonatal intensive care units (NICUs). However, the individual immaturity of vulnerable infants and the need for intensive care measures (e.g. vascular catheters, ventilation) are associated with a high risk for healthcare-associated (HA) infections. The overall incidence of bloodstream infection (BSI) in VLBWI ranges from 10 to 20%, while the rate of Gram-negative sepsis is 3–5% [1]. In VLBWI, 20% of intra-hospital deaths are causally linked to HA infections [2, 3], while survivors are susceptible for long-term sequelae, e.g. chronic lung disease as well as neurocognitive and behavioral deficits [4]. Hence, there is an urgent need to optimize prevention strategies against HA infections and to provide more evidence on infection control bundles.

The period of highest vulnerability for HA infections is between day 7 and day 28 of life. This critical time frame is characterized by a dynamic colonization with hospital-endemic flora and frequent exposure to antibiotics [5]. It has been demonstrated that bacteria colonizing infants might be concordant with invasive pathogens or even starting points of outbreaks [6]. Following several infection outbreaks in German NICUs, the German Commission on Hospital Hygiene and Infection Prevention at the Robert-Koch Institute (KRINKO), Berlin, decided to recommend weekly colonization screening with rectal and pharyngeal swabs for infants requiring intensive care. This screening should be performed according to local microbiological standards in order to detect the following pathogens:

KRINKO I– all multi-drug resistant organisms (MDRO), i.e. Multidrug-resistant Gram negative bacteria (MRGN), 2MRGN (resistant to ureidopenicillins, third generation cephalosporins), 3MRGN (resistant to ureidopenicillins, third generation cephalosporins and fluorchinolones) and 4MRGN (resistant to ureidopenicillins, third generation cephalosporins, fluorchinolones, and carbapenems); methicillin resistant Staphylococcus aureus (MRSA).

KRINKO II– Acinetobacter spp., *Klebsiella pneumoniae*, *Methicillin-susceptible S. aureus (MSSA)*.

KRINKO III– pathogens with high epidemic potential (nosocomial transmissibility) but not MDRO including *Serratia marcescens*, *Pseudomonas aeruginosa*, and Enterobacter spp.

Based on positive screening results proactive hygiene interventions are to be implemented in daily care. This strategy aimes to (a) adjust anti-infective therapy for suspected sepsis in colonized infants, (b) set up an early warning system for outbreaks, (c) prevent spreading of MDRO or pathogens with high epidemic potential by extended barrier precautions and (d) benchmark local epidemiology of MDRO [7]. According to KRINKO recommendations, NICU treated infants who are colonized with 2MRGN should be treated with barrier care (protective disposable gloves and long-sleeved gowns with cuffs during any patient contact) in addition to hand desinfection. However, the implementation of these extended hygiene measures should be effective to prevent HA infections and their deleterious consequences which has not yet been clearly shown [3, 8]. In addition, side effects of barrier protection need to be considered, e.g. reduced patient contact time, decreased quality of hand disinfection as well as economical and ecological burden of single-use of hygienic items.

### The rationale for a cluster-randomized controlled non-inferiority study

The clinical equipoise for this randomized controlled trial (RCT) is based on a potential benefit of extended barrier precautions for reducing HA infection risk and outbreaks in vulnerable babies versus negative effects of potentially non-effective extended hygiene measures. Specifically, a retrospective cohort study including 337 infants suggested that the combined use of alcohol-based hand rub and gloves leads to a 2.8 fold reduction of HA infections [9]. Using a hand hygiene protocol with hand washing, hand rub and gloves a single center study including 200 preterm infants born at 32–36 weeks of gestation reduced HA infections from five to zero cases over a 14-month observation period [10]. In a prospective trial including 120 preterm infants, Kaufman [11] noted a 17% reduction of Gram-positive infections and 64% fewer central line-associated bloodstream infections when non-sterile gloves were used as compared to hand disinfection alone. One of the largest efforts in adults, the Benefits of Universal Glove and Gown (BUGG) cluster randomized trial, found non significant effect of universal glove use on preventing infections with MDRO [12]. Hence there is a need to evaluate the potential benefits of extended precautions and balance against negative effects. Since the introduction of routine colonization screening in 2013, obervational data from German NICUs revealed no significant changes in infection rates and sepsis mortality caused by pathogens included in the screening [3, 13]. While the rate of culture-proven sepsis with Gram-positive bacteria had already been declining in centers participating in a large German cohort study (German neonatal network, GNN) before the screening guideline, the infection rates with Gram-negative pathogens and MRGN remained constant, i.a. 2.5-3%, and < 0.5%, respectively [3]. These data provide the key argument for the non-inferiority design of the our trial.

The following drawbacks of screening and extended barrier precautions exist. First, glove use can be a key barrier to appropriate hand hygiene [14, 15]. In a recent feasibility trial involving 750 infants, Khan et al. [16] found higher hand hygiene compliance rates in standard care time periods as compared to glove use periods at different moments of care: before patient contact or clean procedures as well as after body fluid or direct patient contact. Glove use may therefore confer a false sense of security, as pathogens easily contaminate gloves without performing hand hygiene properly. Second, our cohort studies demonstrated an overuse of reserve antibiotics such as carbapenems [3, 17] associated with implementation of the weekly colonization screening in Germany. Third, extended hygiene measures are time and cost-intensive and may interfere with other priorities of neurodevelopmental support and family-centered care, e.g. avoidance of co-bedding or skin-to-skin contact of twins with different screening results. Fourth, disposable gloves are the most commonly used single-use item in intensive care. Their production requires natural resources, and they significantly contribute to hospital waste [18]. To our best knowledge, there is no evidence on particular benefits on the use of gowns in the care of NICU patients [19].

In the BALTIC trial (Barrier protection to lower transmission and infection rates with Gram-negative 2-MRGN in preterm children), we aim to overcome the limitations of observational studies by a multi-center cluster-controlled design with cross-over after 12 months. This will account for confounding factors such as the number of infants enrolled per year and center-specific effects. In addition, we will promote infection control by on-site trainings and team sessions to promote goal settings and compliance audits on hygiene precautions before the study and in regular intervals. Participating study sites were randomized after they achieved highly reliable performance of existing evidence-based infection prevention practices in on site audits. The following hypotheses will be tested:

1. The care of NICU-treated infants with 2MRGN colonization with standard infection prevention and control stategies (IPC) alone (intervention) is not inferior to standard hand hygiene combined with the use of protective gowns and single-use gloves (control) in terms of the rate of HA infections caused by with Gram-negative pathogens (primary endpoint).
2. Participation in a network study on the above-mentioned topic, including training and basic hygiene audits, increases the compliance to common IPC practices in participating study centers.

The BALTIC trial can add new data to current uncertainties. The multi-center network structure may generate population-representative data on the prevalence of MRGN in German NICUs. BALTIC also provides the basis for implementing a multi-center antimicrobial stewardship program. We also aim to evaluate the costs of extended hygiene measures given the increased consumption of disposable items.

### Benchmarking by analysing single center data

For the design of the Cluster-RCT we evaluated single center data of routine colonization screening (1x/week rectal swab and pharyngeal swab) from VLBWI born between January 1st, 2013 until December 31st, 2017. The clinical characteristics of our cohort including 319 infants is summarized in Table 1.

**Table 1:** Clinical characteristics of single center cohort of very-low-birth weight infants

Clinical parameter		cohort *n* = 319
Sex, female, *n* (%)		160 (51)
Multiple birth, *n* (%)		114 (36)
Gestational age, wk, median (IQR)		28,5 (4,1)
Birth weight, g, median (IQR))		1091 (564)
Mode of birth, *n* (%)	Spontaneous	20 (6)
	Elective casearean	256 (80)
	Emergency Caesarean	43 (14)
Cause of preterm birth, *n* (%) Multiple causes possible	Preterm labor	194 (44)
	Pre-eclampsia	39 (9)
	Pathological Doppler	112 (25)
	Placental abruption	4 (1)
	Intrauterine growth restriction	63 (20)
	Suspected or proven amniotic infection	144 (45)
Duration hospital stay, days, median (IQR)		63 (41)
Antibiotic days/100 days, median (IQR)		13,1 (14,9)
Probiotic use, *n* (%)		174 (55)

**Fig. 1:**
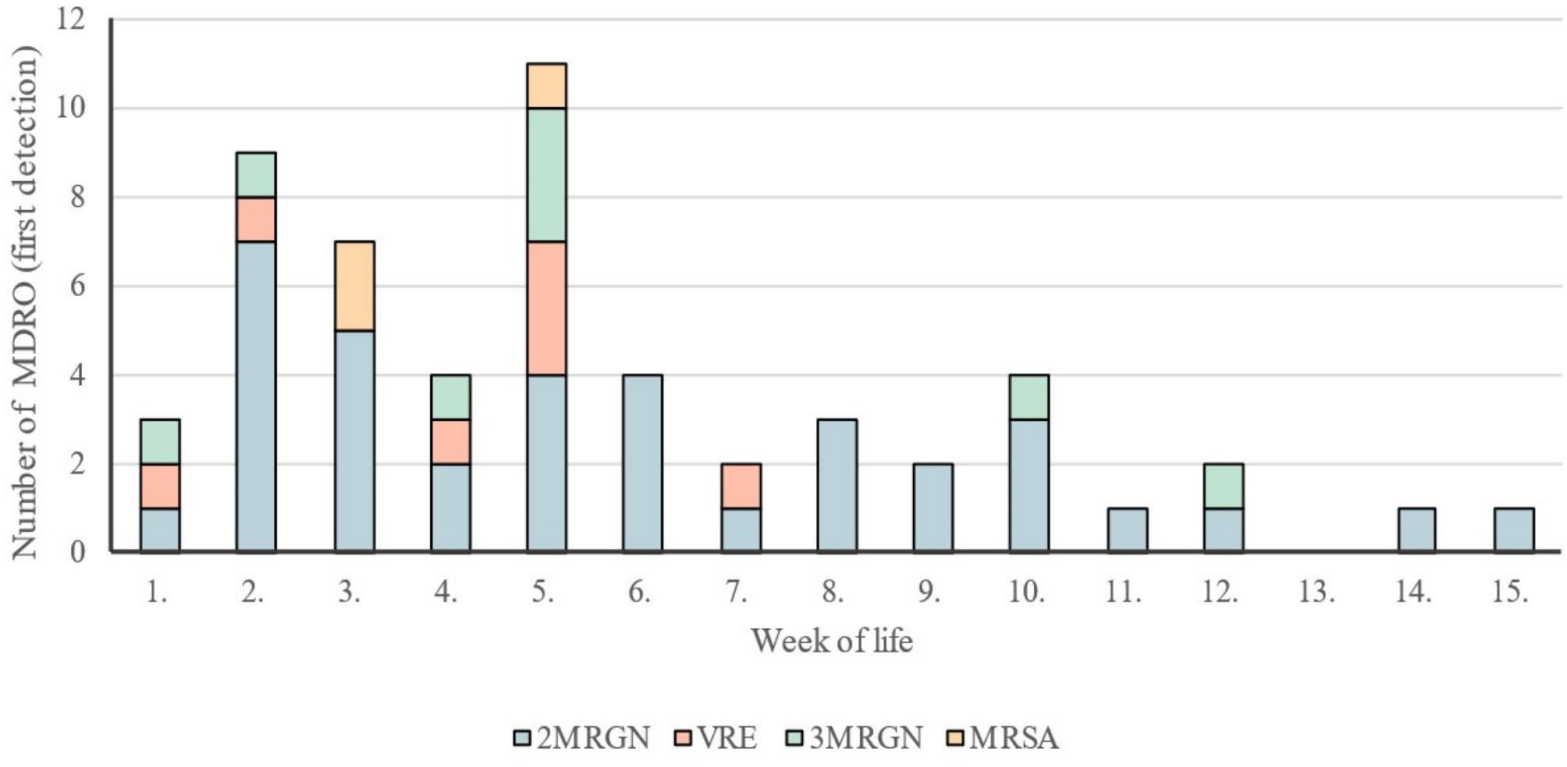
First detection of MRGN, MRSA, MRSA or Vancomycin resistant enterococci (VRE). MRDO = Multi-drug resistant organisms; KRINKO = German Commission on Hospital Hygiene and Infection Prevention, MRGN = Multidrug-resistant Gram negative bacteria, 2MRGN (resistant to Piperacillin and Cefotaxim or Ceftazidim), 3MRGN (resistant to Piperacillin and Cefotaxim or Ceftazidim and fluorchinolones), MRSA = methicillin-resistant *Staphylococcus aureus*, VRE = Vancomycin resistant *Enterococci*. Additional first detections after the 15th week of life: *n* = 4 2MRGN pathogens

Out of 319 infants, 47 (14.7%) were colonized with an MRGN pathogen, of which 30 infants (9.3%) tested positive for a 2MRGN pathogens. One infant was colonized with 2MRGN *E. coli* at birth, 15 infants were colonized within the first 5 weeks of life (Fig. 1). The 2MRGN pathogens were isolated from pharyngeal swabs (*n* = 12), rectal swabs or fecal samples (*n* = 19) or conjunctiva (*n* = 1). Four infants were colonized with > 1 MDRO including two infants with MRSA and 3MRGN *E.coli* and two infants with 2MRGN *Enterobacter cloacae* and either 2MRGN *E.coli* or 2MRGN *Klebsiella pneumoniae*. Colonization with 2MRGN occurred at a median of 33 days. Figure 1 describes the timepoint of first colonization with MDRO in vulnerable infants. Seven infants were initially colonized with antibiotic susceptible bacteria and acquired 2MRGN features during hospital stay.

Out of 319 of infants, 296 received antibiotic therapy for a median duration of 7 days, 252 infants were already started on antibiotics on day 1 of life. MDRO colonized infants were treated for a median of 7 days. Among 2MRGN colonized infants, one infant developed a 2MRGN *E.coli* sepsis on day 6 of life, two infants were diagnosed with 2MRGN sepsis (*Enterobacter* spp.) between day 7 and 28 of life and five infants were treated for culture-negative sepsis. Our data are in line with previous single center studies in Germany with evidence of MDRO colonizing infants in 4.9% of 635 neonates, 12.7% of 584 screened neonates and 15.3% of 1407 preterm infants, respectively [20–22]. In a recent multicenter RCT on the effect of probiotics to prevent MDRO colonization in preterm infants 28–32 weeks of gestation, we confimed center-specific MDRO colonizing rates between 5 and 20% [23]. In the BALTIC trial, we therefore estimated the number of 2MRGN colonized infants to be 10% during the hospital stay.

### Trial design

**Fig. 2:**
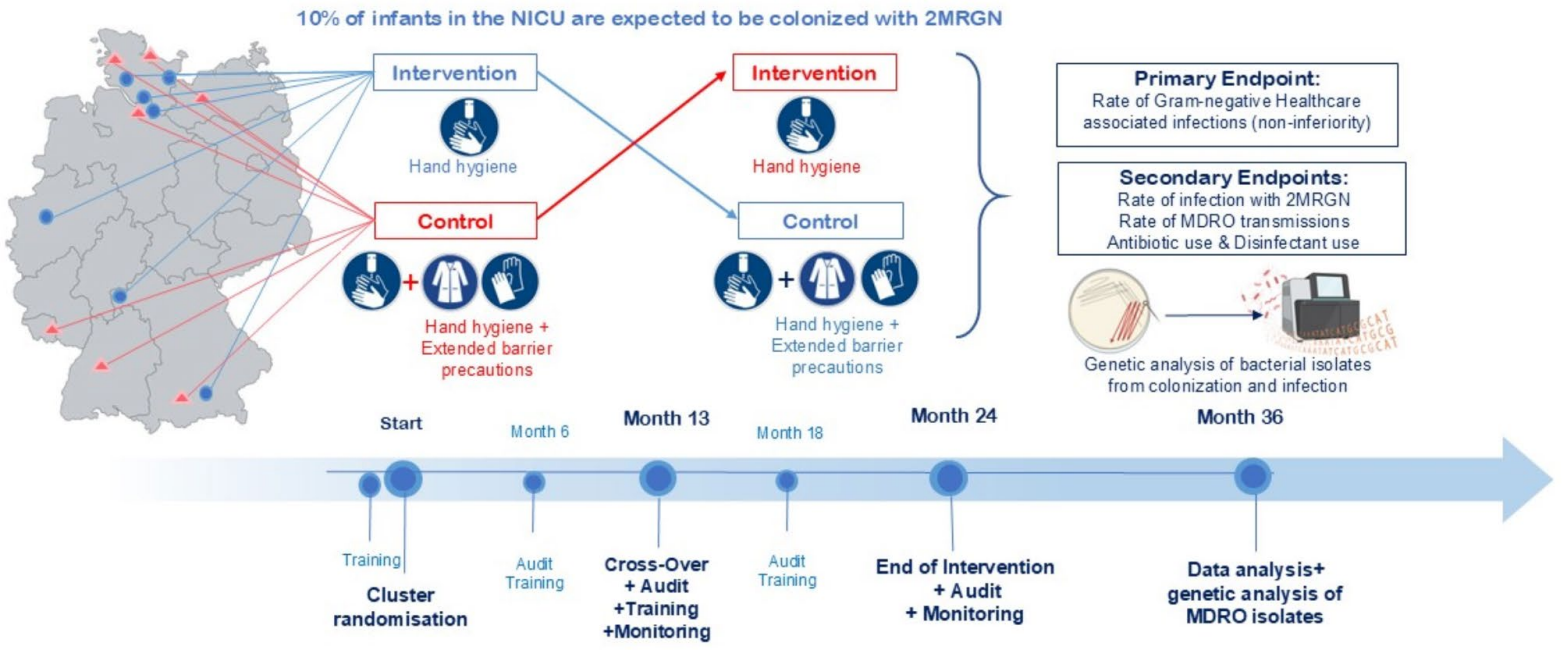
Study design of the BALTIC trial

The study design is depicted in Fig. 2. In the participating centers, all newborns requiring intensive care undergo colonization screening based on KRINKO recommendations. If a 2MRGN positive pathogen is detected this infant is treated with hygiene measures as described in the intervention (focus on standard hand disinfection) or control procedure (standard hand disinfection plus barrier precautions), depending on the randomization of the center. The study has been conducted in a cross-over design with a change between the intervention and control group after 12 months. All other patients (screening negative or positive for a pathogen included in the KRINKO recommendations apart from 2MRGN) receive the standard care based on screening results and local guideline. After 6, 12, 18 and 24 months, the compliance will be evaluated by the means of random observations of hygiene in the NICU in collaboration with local institutions for hospital hygiene followed by standardized training modules. The PICO question of this multicenter, cluster RCT with cross over is whether in the **P**opulation of preterm infants and critically ill neonates requiring intensive care who are colonized with 2MRGN the **I**ntervention ***standard hand hygiene disinfection*** is non-inferior to the current KRINKO recommendation (**C**ontrol) of ***standard hand hygiene disinfection + barrier precautions (gloves/gown)*** for the **O**utcome overall rate of HA Gram-negative bloodstream infections (primary endpoint).

### Site selection and randomization

In total, 12 centers (neonatal intensive care units with level 1 care) were included in the study. Randomization was performed at the level of the individual center, and sites were randomly allocated to start with either intervention or control group with after on-site training and audit in hygiene measures. For site selection, we synergistically used our collaborative networks of the GNN and the PRIMAL study which proved reliability in previous clinical studies and enables to compare data on infections before during the BALTIC trial. Background information about the participating sites were collected through a structured interview, followed by a baseline observation, hand hygiene training and audit. Hand hygiene training included presentations on hand hygiene compliance in NICUs and room for improvement, guidelines on good hand hygiene practice and other measures of infection control, consequences of HA infections and outbreaks, distribution of flyers/posters related to the WHO’s “5 Moments forHand Hygiene” [24] and access to videos/webinars/e-learning. The training was organized by the clinical project management team in Lübeck, in collaboration with the respective hospital hygiene leadership and the hygiene officers (Link Nurses) / physicians.

Further requirements for participation in the study are (a) implementation of an admission screening and a weekly colonization screening as recommended, (b) availability of a microbiological laboratory that collects antimicrobial resistance data and supports further measures in the event of a suspected HA infection outbreak, (c) support from the local institutions for hospital hygiene for hygiene training and audits and (d) support from the local pharmacies and health business departments which provide data on antibiotic consumption and gown/glove use.

### Audits

Quality controls and data security are ensured by the availability of a study protocol, by a monitoring visit prior to the start of the study and by standard operating procedures (SOP, e.g. for diagnosis of sepsis, transmission). Audits are initiated to verify adherence to the study protocol and ICH-GCP (Good clinical practice) procedures.

In order to evaluate the compliance with infection control measures, direct observations were performed on a random basis by the study team and IPC nurses. The observation duration ranged from 20 min to a maximum of 2 h per day, ensuring that other treatment priorities, e.g. kangaroo care, remain unaffected. We aimed for anonymous documentation of disinfectant use (nurse, doctor, service staff, parents, others), and compliance with 5 moments of proper hand hygiene, use of gloves and long-sleeved gowns. The Hawthorne effect, i.e. alteration of typical behavior by being observed and variability among observers (external representatives of the study leadership, local hygiene experts, and link nurses) are potential disadavantages of the direct observation approach [25]. As a second method we therefore collected data on disinfectant use in the clusters (mL per patient day) which can overcome the Hawthorne effect but only serves as a surrogate fo hygiene compliance.

Management of transmissions and possible outbreaks were at the discretion of the local team based on NICU guidelines which was different across the NICUs. The local standards and possible changes were recorded at three time points: initiation of the study-centers, at after 12 months (cross-over) and at the end of the study after 24 months. In the manuscript reporting data we will also report on extended hygiene measures and additional tests for outbreak identification (e.g., genotyping, pulse field electrophoresis).

### Outcomes

The primary endpoint is the rate of HA bloodstream infections with Gram-negative pathogens in all preterm and newborn infants who received colonization screening. This outcome is defined clinically according to NEO-KISS criteria (at least two clinical signs of sepsis + one laboratory sign, e.g. increase in C-reactive protein > 10 mg/l) after 72 h of life and requires the detection of a Gram-negative pathogen in the blood culture. Blood-culture isolates will be sent from the study centers to the study management.

Key secondary endpoint is the 2MRGN transmission rate which is assumed if another infant is colonized with the same 2MRGN pathogen after 2MRGN colonization or infection of an infant requiring intensive care in a temporal (maximum 90 days) and spatial context (potential contact in the intensive care unit) [26]. This assumption is made if, in addition to pathogen identification, the antimicrobial susceptibility testing patterns are identical. Suspected transmission of or bloodstream infection with an 2MRGN pathogen will be reported to the study management as severe adverse event within 24 h.

Transmission/outbreak management remains the responsibility of the local interdisciplinary infection control team and is carried out according to local standards, including the decision whether to initiate genetic identity testing in the acute situation. 2MRGN isolates will be shipped from local sites and molecular typing will be performed at the Departemnt of Infectious Diseases and Medical Microbiology at the University of Lübeck. Other secondary outcomes at the level of study sites per period (“study phase”) are:

- Rate of clinical and culture proven infections.
- Rate of infection outbreaks.
- Number of initiated antibiotic cycles.
- Duration of antibiotic treatment (days).
- Exposure to reserve antibiotics, including Carbapenems per study site in % infants.

To assure documentation of relevant outcomes, intensive interdisciplinary collaboration (neonatology/infectiology/hygiene/pharmacy/management) is required. Data are collected by monthly cluster report forms on infection and transmission rates and yearly surveillance of antibiotic use, disinfectant consumption, and cost estimations.

### Stopping rules

The coordinating investigator has the right to discontinue the study in centers that experience insufficient study conduction, technical-logistic problems and repetitive outbreak situations that require extended barrier precautions in all patients, which would interfere with the study questions. The study sites are instructed to discontinue the study only if substantial problems occur. The coordinating investigator will be informed immediately, should ethical or safety concerns occur at any study site.

### Quality assurance and monitoring

During the study, quality assurance measures including supervision by authorities are implemented. All study centers have agreed that a study monitor will review the data collection before, during and after the study to ensure that the study is conducted in accordance with the protocol, with SOPs and the corresponding regulations of GCP. In addition to the monitoring procedures, audits will be carried out in accordance with the ICH-GCP Guidelines. In the context of an audit, the planning, execution, and analysis of the study are checked to evaluate whether all study processes are conducted in accordance with the German law or the ICH Guidelines. This includes the storage of data and the organisation of the study centres, the original documents in accordance with data management agreements between sponsors and participating centres. The objective of audit measures is to ensure that all results and conclusions that ultimately appear in the final report are readable from the raw data.

### Data management

The primary data set is generated and managed by the clinical project management of the study management (K.F., C.H.). The data generated on the basis of the CRFs are entered into the central database at the study management using the “double-entry” principle. To ensure optimal data protection, the data on settlement or primary endpoints are stored and evaluated without any reference to the name of the child or the personal data of the parents. Pathogen isolates are stored without patient identifying data but date of collection to allow a temporal and cluster-related assignment (retrospective detection of transmission by central biobanking and pathogen genotyping). The provisions of the Data Protection Act are strictly adhered to, ensuring the data is safeguarded from third-party access.

### Sample size calculation and statistical analysis

GNN and PRIMAL centers treat on average 150 preterm and critically ill neonates per year with compulsory colonization screening. The center-specific rates of Gram-negative HA infection ranges between 1.5 and 6%. Based on single center data as described above and other cohort studies [20–22] we expect 10% of these infants to be colonized with 2MRGN during hospital stay, i.e. 180 infants per 12 months observational period.

Based on previous data, we assume an intracluster correlation of maximally 0.01. Given that the observed correlation is very low, the time lag between the two trial periods is long and different patients are included in the two trial periods at each site. No carry-over effects are assumed, as we are not expecting that effects of one treatment period would influence the second period (after cross-over) since medical care-givers are aware of the study period they are in. Date from two periods at one site are regarded as two independent sites. We further assume a frequency of Gram-negative HA infections in the controls of 3% and a non-inferiority limit of 5%. Striving for a power of 0.8 (1-ß) with a one-sided significance level α of 0.05, at least *n* = 18 patients per site and phase are required for analysis with 12 sites, thus *n* = 432 in total. To allow for a maximal drop-out of 10% drop-out, at least 480 patients need to be included.

The primary data analysis will be performed according to the intention-to-treat principle (non-linear mixed effect regression models, logit link). Generalized estimating equations are performed to determine the infection rates between the two regimens. Secondary endpoints are analysed as the primary endpoint per individual or per cluster.

## Results

The first center was randomized in October 2020, the last center was randomized in June 2021. In total, 18 sites were screened for participation, 12 sites met all requirements for recruitment and randomization, 6 were allocated to intervention and 6 were allocated to control with cross-over after 12 months. Data collection was completed in January 2024. Regular trainings on infection control measures and audits were regularly performed on-site or by remote meetings during the lockdowns in 2020/2021. Data analysis is ongoing and will last until December 2024.

### Data dissemination

After completion of the study, the results will be presented at national and international congresses and published in peer-reviewed journals. The results are also presented in the media of the parents’ associations (Federal Association “The Pregnant Child” and EFCNI). The public is informed about the study in the form of press releases and background articles on preterm birth and infection risks.

### Ethics

The BALTIC study was approved by the institutional review board of the University of Lübeck (primary vote, 19–275) and by the review boards of all participating sites (secondary vote). No identifying data about the infants were collected.

### Funding

The study was funded by the German Ministry of Education and Research (German Center for Infection Research), the German Society for Pediatric Infectious Diseases and the Damp Stiftung.

## Discussion and conclusion

The BALTIC study was drafted to create evidence on the effectiveness of extended barrier precaution measures in critically ill newborns being colonized with MDRO. Current guidelines recommend regular screening and the consequent use of gloves and gowns in case of 2MRGN colonization, however, the evidence on clinically relevant outcomes is scarce. Potential disadvantages of extended barrier precautions are time- and cost intensity and may interfere with individualized neurodevelopmental care. The multicenter design of BALTIC creates new insights into center-specific endemic flora of NICUs and population-representative data on MDRO colonization dynamics. Standardized trainings on the proper use of infection control measures will lead to an improved awareness on preventive strategies and the establishment of network platforms including antibiotic stewardship programs. This study also has limitations. Training and audits were performed as controlled co-intervention, however, significant variation in compliance due to Hawthorne or observer bias and irregular attendance of healthcare professionals for training lessons cannot be prevented. Considering that there are few studies that have rigorously investigated the current recommendations for infection control in neonatology, BALTIC will contribute to more refined and successful interventions in the future.

## Electronic supplementary material

Below is the link to the electronic supplementary material.

Supplementary Material 1

## Data Availability

The full protocol is registrated in the German registry of clinical studies (DRKS, Trial registration number: DRKS00019103), we plan to grant access to the full dataset.The datasets used and/or analysed for the benchmarking results are available from the corresponding author on reasonable request.

## Declarations

### Trial registration number

DRKS00019103.

## Abbreviations

BALTIC: Barrier protection to lower transmission and infection rates with Gram negative 2-MRGN in preterm children
BUGG: Benefits of Universal Glove and Gown Trial
BSI: Bloodstream infection
GCP: Good clinical practice
GNN: German Neonatal Network
HA: Healthcare-associated infections
IPC: Infection prevention and control
KRINKO: German Commission on Hospital Hygiene and Infection Prevention at the Robert-Koch Institute
MDRO: Multidrug-resistant organisms
MRGN: Multiresistant gram-negative bacteria
2MRGN: MRGN bacteria with *in-vitro* resistance to piperacillin and 3rd generation cephalosporins
MRSA: Methicillin resistant Staphylococcus aureus
NICUs: Neonatal intensive care units
RCT: Randomized controlled trial
SOP: Standard operating procedure
VLBWI: Very low birthweight infants: birth weight < 1500 g
VRE: Vancomycin resistant *Enterococci*
